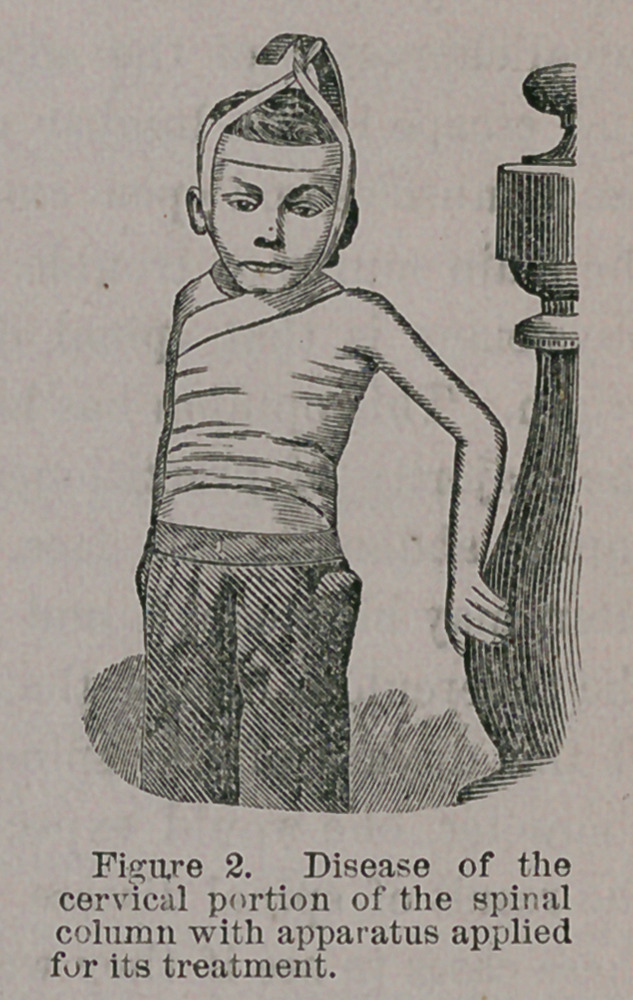# Clinical Remarks upon Surgical Cases Occurring in the Sisters of Charity Hospital

**Published:** 1875-03

**Authors:** Julius F. Miner


ART. III.—*Clinical Remarks upon Surgical Cases occurring in the Buffalo Hospital of the Sisters of Charity. By Prof. Julius F. Miner, M. D.* Reported by W. W. MINER, M. D.

CASE XVI.—*Angular Curvature.*—The diseased condition which is the origin of the deformity noticeable in the back of this child, is known as angular curve or Pott's disease of the spine. The deformity is in the upper part of the dorsal region, and is unfavorably located as far as treatment is concerned. It cannot be perfectly restored, and you should not be persuaded otherwise. Writers on this subject have claimed more than facts will warrant. Apparatus has been recommended, which is constructed with a hinge, placed opposite the angle of the spine, and the design of it is to force a restoration of the vertebræ to their normal position,—the obliteration of the angular deformity. It attempts too much. The deformity is not capable of being perfectly removed, if any essential change has taken place in the vertebral substance. There is here also a degree of paralysis of the lower extremities; it has been noticeable for a month or two. Such condition is quite a frequent accompaniment in the course of the disease. It was formerly supposed that this paralysis arises from pressure upon the spinal cord by the displaced vertebræ. It is now known that it is due to an inflammatory condition of the cord, or of its membranes, rather than to direct pressure upon the nerve substance. It generally disappears in due time, and that while the curve has been in no degree restored, and it rarely leaves any permanent ill effects. I had a patient with disease of the spine at the General Hospital some time ago, who was paralyzed for a whole year, but is now actively employed in a grocery story in this city.

An abscess is sometimes formed in connection with the diseased structures, and the pus formed may burrow in various directions, depending upon the location of the spinal disease and the anatomical character of the adjacent soft parts. It accordingly may find escape in the lumbar or cervical regions, or may follow the psoas muscles and open externally at a point quite remote from the main source of trouble. The generally entertained opinion of physicians is that spinal disease is of scrofulous or tuberculous origin. This opinion has long been held, and is not questioned by the majority of practitioners. The patients may present a large, tumid abdomen, pale face, thin muscles, anemic condition, and later, may have cough and consumption, but it seems to me that the tuberculous disease that may develop is not the primal source of the disease of the spine. If this is of itself tuberculous in character, one would expect that death would sooner or later be the result of spinal disease. I have for ten or fifteen years watched these cases to see if they were fatal, and have not found the first instance of this kind that would confirm the theory of tubercular origin. I am of the opinion that the disease is not tubercular or scrofulous in character, but that it is of the ordinary character presented by disease of joints and bones.

Though not susceptible of perfect restoration, the cases are greatly amenable to treatment. Local support is required, to relieve pressure and steady the natural movements; it will prevent farther progress of the deformity. Physicians all over the country are prescribing cod liver oil, iodide of potassium, etc., for this affection, and think that they are then doing well enough. These are good remedies to be sure, but they are not curative of the real trouble. It is doing next to nothing to give these patients medicines alone and tell them to take their chances. Care in treatment, local support, recumbency, rest, cod liver oil, iodides, tonic remedies, out door air, and all that the patient can eat, are necessary in the treatment of this disease. Any good physician can order or contrive appliances that will generally mitigate and relieve the case he may have. The idea is to relieve the pressure that naturally comes upon the structures which have become involved in disease, and are no longer fit for their natural use.

Figure 1. Apparatus of ordinary construction used for Pott's disease affecting the dorsal or lumbar region of the spinal column.

Figure 2. Disease of the cervical portion of the spinal column with apparatus applied for its treatment.

An apparatus such as I show you, of firm material with a padded covering is made, having a stiff back, moulded to the peculiar shape of the spine, in the case for which it is intended, provided at its lower part with pelvic band that may be approximated closely to the pelvis and thus form a basis of support, while by means of arm pieces of elastic material projecting in like manner above, support may be given the shoulders or head, which will very greatly remove the weight of the upper portion of the body from the opposed surfaces of diseased vertebræ.

This relief of weight and pressure is a very valuable remedial agent, will generally prevent progress of disease and deformity, and greatly relieve the patient. We need not say much about bringing the parts back to their natural position and removing the deformity; it is quackery, I think, to talk thus and propagate such views. Many things have been written that I do not believe were done, or at least results are frequently to be inferred from published writings that it is impossible to obtain. If, as I have said, ulceration or absorption of bone has taken place, deformity is permanent. Possibly, deformity from disease of the interverte-bral cartilages may be overcome.

You can put your patients in bed for a time, thus relieving the pressure upon diseased surfaces and affording you an opportunity to obtain suitable apparatus, which allows the patient to be about and obtain out door exercise. An appliance obtained from an ordinary unskilled instrument maker has been used in this case, and as in general, the instrument thus obtained is of no use whatever. An intelligent understanding of the principles to be carried out in the use of the external appliances, and an adaptation of them to the case in hand, is thoroughly essential to success in treatment.

The friends of this child have been told by the physician, who has had her in charge, that her paralysis is permanent—that the child will never walk. The fact is, however, that while active progress of the disease is taking place, while acute symptoms are present, the paralysis occurs, and afterwards it disappears. Very rarely is the paralysis permanent. The deformity is not capable of being effaced, but may be arrested in its progress, and circumstances being favorable, much may be expected from natural processes of repair. No injury that would account for the occurrence of disease in the present case is known of, though such cause is to be sought for by physicians, and is often overlooked by persons having children in charge. Injury of this kind is oftener recognized where the patients are more advanced in years and are in the habit of looking after themselves.

Those who are thus brought to you unable to walk, and supposed to be near death from scrofulous or tuberculous disease, will often after a while walk, grow sound in health, and in advanced years be active and efficient in the ordinary affairs of life.

## Figures and Tables

**Figure 1. f1:**
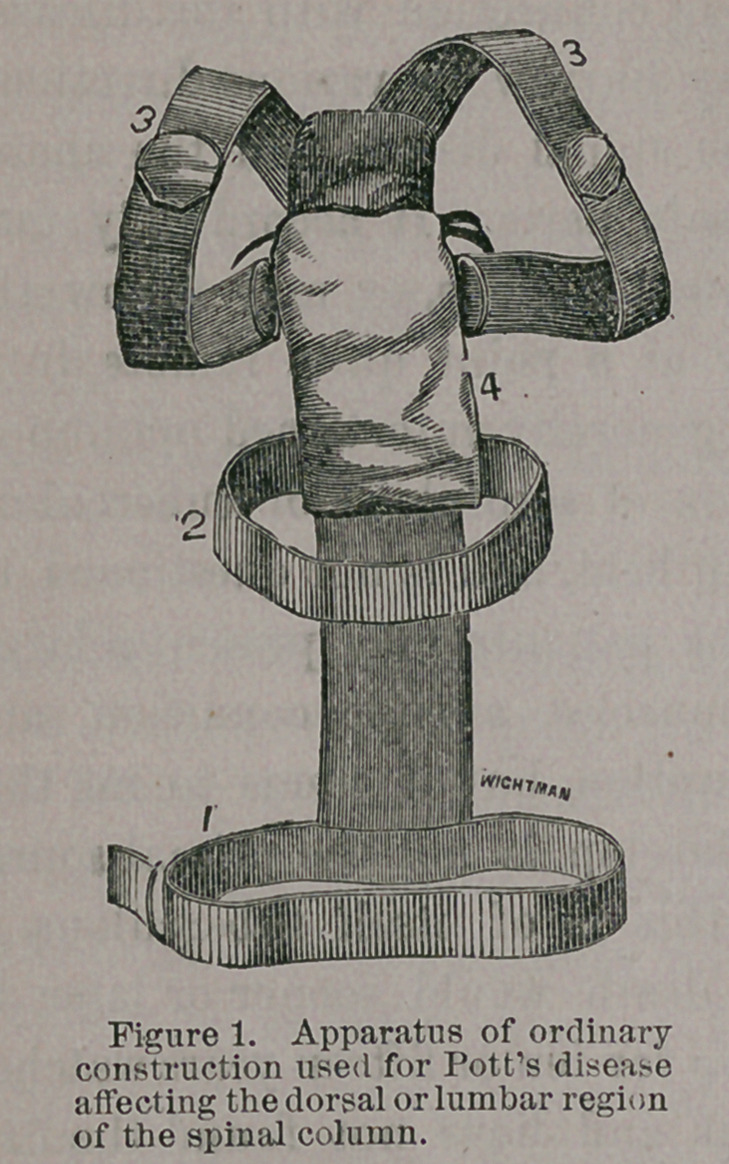


**Figure 2. f2:**